# Azygos Vein Lead Implantation For High Defibrillation Thresholds In Implantable Cardioverter Defibrillator Placement

**Published:** 2010-01-07

**Authors:** Naga VA Kommuri, Sri Lakshmi S Kollepara, E Saulitis, MA Siddiqui

**Affiliations:** Sinai-Grace Hospital

**Keywords:** Azygos vein lead, High defibrillation threshold, Implantable cardioverter defibrillator

## Abstract

Evaluation of defibrillation threshold is a standard of care during implantation of implantable cardioverter defibrillator. High defibrillation thresholds are often encountered and pose a challenge to electrophysiologists to improve the defibrillation threshold. We describe a case series where defibrillation thresholds were improved after implanting a defibrillation lead in the azygos vein.

## Introduction

Implantable Cardioverter defibrillator (ICD) has evolved as an important therapy to prevent sudden cardiac death. Early experience with ICDs revealed cases of sudden death in patients due ineffective defibrillation as a result of inadequate defibrillation thresholds (DFT) safety margins [1-4]. Subsequently it has been a standard of care to check the DFTs when a new ICD is placed. DFT is defined as the lowest amount of energy delivered by the ICD to successfully terminate two episodes of induced ventricular fibrillation. DFTs can be measured by multiple techniques but a safety margin of 10 joules for defibrillation is standard of care [5-9].

High DFT is defined as a DFT value less than 10 joules lower than the maximum out put of defibrillator generator. Studies have demonstrated that a significant percentage of patients (6 to 16 %) have higher DFTs [3]. DFTs can be reduced by multiple mechanisms. We describe a case series of 3 patients where leads were placed in azygos vein to improve defibrillation threshold.

## Case 1

An 88 year old male with a history of dilated cardiomyopathy, severe left ventricular dysfunction (Ejection fraction of 30 %) with intraventricular conduction delay had symptomatic heart failure in spite of being on maximal medical therapy and a cardiac resynchronization therapy with defibrillator back up was recommended as he was functional at base line.

**Figure 1:**
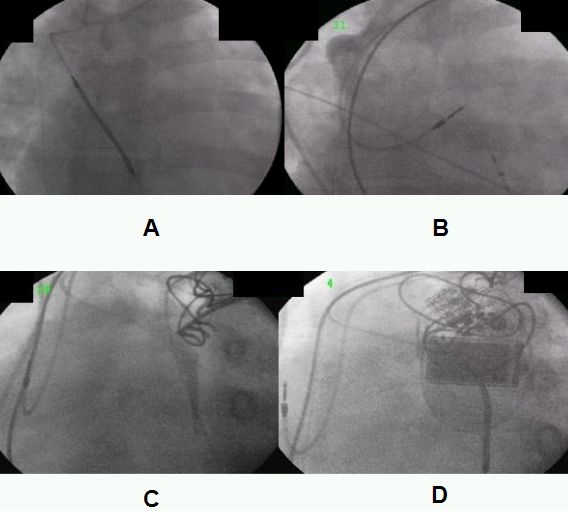
A. Transvenous lead in azygous vein; B. RAO view showing origin of azygous vein; C. Posterior course of azygous vein; D. Lateral view of azygous vein

**Figure 2:**
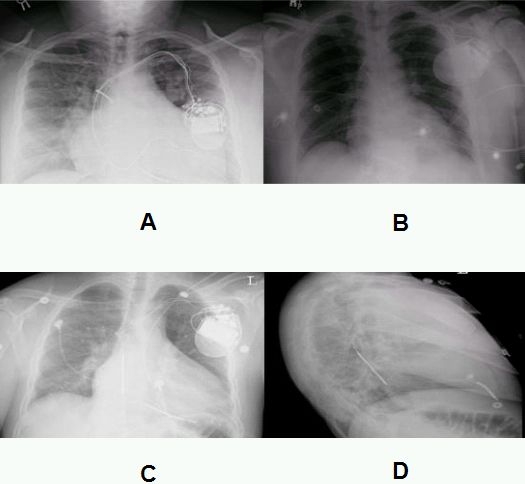
Chest X-ray showing the position of azygous vein lead

After informed consent was obtained, patient was prepared and draped in standard sterile fashion. Venous access was obtained through left axillary vein by modified seldinger technique, introducers were placed, leads were advanced and positioned in the right ventricular apex (0181 ICD lead Guidant), right atrial appendage (4086 Guidant) and via the coronary sinus in the posterior lateral aspect of the left ventricle (4517 Guidant). After demonstration of appropriate pacing sensing function, the leads were anchored to pectoralis muscle and connected to a Guidant pulse generator (H 219). The leads along with pulse generator were placed inside the prepectoral pocket and the defibrillation threshold was assessed. There was difficulty in defibrillating at initial polarity and reversed polarity at 21 and 31 joules. A separate Superior vena cava (SVC) coil was placed at the juncture of the SVC and right atrium which did not improve the defibrillation threshold in either polarity. Subsequently the azygos vein was cannulated utilizing a Rapido IC 90, then a long sheath was placed and a Transvene lead (6937 A-Medtronic) was placed in azygos vein and the patient was successfully defibrillated at 31 joules (Figure 1A). Post operative chest x-ray confirmed the position of lead (Figure 2A)

## Case 2

A 42 yr old male with a history of dilated cardiomyopathy and severe left ventricular dysfunction who had previously underwent ICD placement for primary prevention presented after receiving shock for ventricular tachycardia. Upon device interrogation sensing and impedance were within normal limits but telemetry revealed inconsistent termination of ventricular tachycardia. In light of these findings it was felt that adding a shock lead in superior vena cava or azygos vein will help to improve DFT. Prior to the procedure DFT was assessed and 10 joule safety margin was not demonstrated. Vascular access was obtained through the left axillary vein and a Transvene lead (6937 A-Medtronic) was advanced and placed in SVC. Defibrillation threshold was assessed and there was inability to shock the patient both at 21 joules and 31 joules at initial and reverse polarity. Subsequently the Transvene lead (6937 A-Medtronic) was advanced to the azygos vein. Defibrillation was successfully achieved at 21 joules. Post operative chest x-ray confirmed lead position (Figure 2B)

## Case 3

A 30-year-old male with a  history of sickle cell anemia,  left ventricular dysfunction with (EF: 20% to 30%) was found to have episodes of nonsustained ventricular tachycardia. A single chamber ICD was recommended as a primary prevention against sudden cardiac death. After informed consent vascular access was obtained using modified seldinger technique and right ventricular lead was placed and then secured to ICD pulse generator. Defibrillation threshold was assessed and there was inability to shock the patient both at 21 joules, 31 joules and 41 joules in initial and reverse polarity. DFT did not improve even after placing Transvene lead (6937A -Medtronic) in the SVC. Subsequently after the azygos vein was cannulated and the Transvene lead (6937 A-Medtronic) was advanced and positioned in the azygos vein defibrillation could be successfully achieved at 21 joules. Post operative chest x-rays confirmed lead position (Figure 2C and Figure 2D)

## Azygos vein Cannulation Technique

After the setting the camera at 25º RAO view a Guidant Rapido IC 90 is advanced and a clock wise torque is performed once the catheter is in the proximal SVC. A 20 cc syringe is utilized with a 50:50 dye mixture and once the distal portion of catheter is in a side branch, venography is performed (RAO/LAO view). After confirming the position of catheter in azygos vein a stiff wire is advanced and Rapido IC 90 is exchanged for a long safe sheath which is advanced and positioned in azygos vein (Figure 1B,C,D). A Medtronic Transvene lead (6937 A) is advanced and positioned in the azygos vein and then the sheath is gently peeled away.

## Results

In all the patients adding a defibrillation coil in the azygos vein helped in achieving adequate defibrillation threshold. The average flouro time was 2 -4 minutes.  There were no procedure related complications and no lead displacement after a mean follow up of 18 months (14-24 months).

## Discussion

The prevalence of sudden cardiac death in USA is estimated to be 400,000 to 500,000 patients annually [6]. ICDs have evolved as a significant tool for the primary prevention of sudden cardiac death and a have been shown to improve survival in patients who are at high risk for ventricular arrhythmias [7,8].

Studies have demonstrated that patients with QRS prolongation in hypertrophic cardiomyopathy, non ischemic dilated cardiomyopathy and patients who are suitable for resynchronization defibrillators may have higher DFTs [10,11]. Earlier studies have also demonstrated the  clinical characteristics that identify high defibrillation thresholds are NYHA (New York Heart Association) Class III, IV, low ejection fraction, no previous history of bypass surgery, prior amiodarone use preoperatively (in the last 6 weeks), and presenting with ventricular fibrillation  [16].

DFTs can be reduced by multiple mechanisms like active can technology, repositioning of right ventricular lead, biphasic shock wave forms, and implantation of subcutaneous arrays [10,14,15]. DFTs using the "active can" might be influenced by the site of implantation and possibly need additional coils to improve DFTs [17]. Subcutaneous array placement can be a technical challenge to the clinician and also can be cause of discomfort to the patient.

The azygos vein in adult is the vessel which persists from the fetal right posterior cardinal and supracardinal veins. It transports deoxygenated blood from the posterior walls of the thorax and abdomen into the superior vena cava.  It usually starts opposite the first or second lumbar vertebrae and is formed by the union of the ascending lumbar veins with the right subcostal vein and enters the thorax through aortic hiatus of diaphragm. Subsequently it ascends in the posterior mediastinum arching over the right main bronchus and root of the right lung and then joins superior venacava before that vessel pierces the epicardium. Studies have demonstrated that as the electrode is located posterior to the heart the shock vector probably better encompasses the mass of myocardium that is posterior to the RV coil, the SVC coil and the casing of the ICD generator [12,13]. Cooper et al demonstrated that the success rate of improving DFTs is similar in both azygos vein leads and subcutaneous leads [13]. This procedure is safe and earlier case series also did not report any complications [12,13].

## Conclusion

In patients with high DFTs azygos vein lead placement is a safe option to lower DFTs. Cannulation of the azygos vein is possible using readily available tools for lead placement and is a less invasive alternative to epicardial shocking lead placement in patients who are difficult to achieve successful defibrillation. Larger studies are needed to further investigate this technique.
